# Children and youth with ‘unspecified injury to the head’: implications for traumatic brain injury research and surveillance

**DOI:** 10.1186/s12982-015-0031-x

**Published:** 2015-06-25

**Authors:** Vincy Chan, Robert E. Mann, Jason D. Pole, Angela Colantonio

**Affiliations:** Toronto Rehabilitation Institute, University Health Network, Toronto, ON Canada; Rehabilitation Sciences Institute, University of Toronto, Toronto, ON Canada; Pediatric Oncology Group of Ontario, Toronto, ON Canada; Centre for Addiction and Mental Health, Toronto, ON Canada; Dalla Lana School of Public Health, University of Toronto, Toronto, ON Canada

**Keywords:** International classification of diseases version 10, Traumatic brain injury, Pediatrics

## Abstract

**Background:**

The case definition for traumatic brain injury (TBI) often includes ‘unspecified injury to the head’ diagnostic codes. However, research has shown that the inclusion of these codes leads to false positives. As such, it is important to determine the degree to which inclusion of these codes affect the overall numbers and profiles of the TBI population. The objective of this paper was to profile and compare the demographic and clinical characteristics, intention and mechanism of injury, and discharge disposition of hospitalized children and youth aged 19 years and under using (1) an inclusive TBI case definition that included ‘unspecified injury to the head’ diagnostic codes, (2) a restricted TBI case definition that excluded ‘unspecified injury to the head ‘diagnostic codes, and (3) the ‘unspecified injury to the head’ only case definition.

**Methods:**

The National Ambulatory Care Reporting System and the Discharge Abstract Database from Ontario, Canada, were used to identify cases between fiscal years 2003/04 and 2009/10.

**Results:**

The rate of TBI episodes of care using the inclusive case definition for TBI (2,667.2 per 100,000) was 1.65 times higher than that of the restricted case definition (1,613.3 per 100,000). ‘Unspecified injury to the head’ diagnostic codes made up of 39.5 % of all cases identified with the inclusive case definition. Exclusion of ‘unspecified injury to the head’ diagnostic code in the TBI case definition resulted in a significantly higher proportion of patients in the intensive care units (p < .0001; 18.5 % vs. 22.2 %) and discharged to a non-home setting (p < .0001; 9.9 % vs. 11.6 %).

**Conclusion:**

Inclusion of ‘unspecified injury to the head’ diagnostic codes resulted in significant changes in numbers, healthcare use, and causes of TBI. Careful consideration of the inclusion of ‘unspecified injury to the head’ diagnostic codes in the case definition of TBI for the children and youth population is important, as it has implications for the numbers used for policy, resource allocation, prevention, and planning of healthcare services. This paper can inform future work on reaching consensus on the diagnostic codes for defining TBI in children and youth.

## Background

Traumatic brain injury (TBI) is “an insult to the brain that affects its structure or function, resulting in impairments of cognition, communication, physical function, or psychosocial behavior”. It includes “open head injuries (e.g., gun shot wound other penetrating injuries) or closed head injuries (e.g., blunt trauma, acceleration/deceleration injury, blast injury)” [1]. TBI is a major public health concern and is a leading cause of death and disability worldwide [2, 3]. A birth cohort study showed that up to 33 % of the population had a brain injury that required medical attention by the age of 25 [4] and recent epidemiological data showed that rates of children and youth with TBI worldwide ranged from approximately 125 to 1,337 per 100,000 [5–8]. This variability can be explained by the wide range of ICD-10 codes that are currently used to define TBI. A recent systematic review found that case definitions ranged from S06.0 (concussion) only to the entire “injury to the head” chapter (S00–S09) [9]. Further, studies that focus only on hospitalization data [10–12] are likely to capture more moderate to severe cases while studies that include emergency department data are likely to capture milder cases that seek medical treatment [5, 6, 8]. As such, it has been suggested that current estimates of TBI are likely underestimates due to the variability in coding and case definitions [13] given that there is current no consensus on the case definition to define TBI. Further, the coding of diagnoses is prone to errors and misclassifications [14–16] and has a tendency to miss milder TBI (mTBI) [17, 18]. It is important to recognize that even a mTBI can have long term consequences, in particular for the developing children and youth population [19]. The current lack of consensus on the case definition for TBI and the validity of various diagnostic codes make it difficult to accurately and appropriately capture information on pediatric TBI that can be used for planning and resource allocation.

A recognized challenge in accurately identifying TBI cases in healthcare administrative data is the use of the ‘unspecified injury to the head’ diagnostic codes (thereafter referred to as ‘unspecified’ codes/cases). The International Classification of Diseases (ICD) is considered the “standard diagnostic tool for epidemiology, health management, and clinical purposes” [20] and are used to identify cases in healthcare administrative databases. Bazarian and colleagues examined the ICD version 9 (ICD-9) case definitions for TBI and found that the ‘unspecified’ ICD-9 codes made up 58 % of the TBI cases, with 62.4 % of these ‘unspecified’ cases being false positives [18]. Shore and colleagues also found that 58 % of the TBI cases in their sample were ‘unspecified’ cases [15]. This finding is of paramount importance as resource allocation and prevention are dependent on the cohort of individuals identified by the case definition. If some of the individuals identified do not truly have a TBI, this can negatively impact prevention efforts and create a misleading understanding of the needs of the TBI population. As indicated, there is currently a wide range of ICD-10 codes used to define TBI [9], including diagnostic codes that do not necessarily reflect a brain injury. However, there is no information as to the extent to which these diagnostic codes affect the number and profile of the TBI population of interest. Specifically, ‘unspecified injury to the head’ diagnostic codes are commonly used; however, there is currently no data that can assist in the interpretation of the resulting incidence and outcome.

To date, most research on the case definition for TBI has focused on the ICD-9 codes. However, the ICD is in its tenth version (ICD-10) and is already used by many countries worldwide [21, 22]. Further, there has been a lack of focus on the case definition specifically for the pediatric population, who are more vulnerable to injury and negative long-term consequences [23] due to the developing brain and cognitive, attention, and executive functions and limited communication abilities [24–27]. Finally, despite the knowledge that including ‘unspecified’ cases in the TBI population reduces the specificity of the case definition for TBI, there is currently no information on the extent to which the profile and outcomes are affected when ICD-10 ‘unspecified’ diagnostic codes are included in the TBI cased definition. This information can assist in the interpretation of current data on TBI.

The objective of this paper is to address the above research gaps by determining the degree to which the number and rate, demographic and clinical characteristics, intention and mechanism of injury, and discharge dispositions of children and youth with TBI are affected by the inclusion of ‘unspecified injury to the head’ diagnostic codes. Results provided can assist in understanding the profile and outcome of individuals with these ‘unspecified’ diagnostic code. This paper will focus on the pediatric population aged 19 years and under in Ontario, Canada, between fiscal years 2003/04 and 2009/10. The availability of accurate information is essential to evaluating, planning, and transforming healthcare systems to better address the needs of this vulnerable population. This study serves to provide evidence documenting the extent to which ‘unspecified’ diagnostic codes influence the numbers and outcomes of the TBI population that can be used as a baseline for future work on reaching an appropriate case definition to define TBI in children and youth that can be used worldwide.

## Methods

### Data source

Cases in the emergency department (ED) and acute care were identified in the Canadian Institute for Health Information (CIHI) National Ambulatory Care Reporting System (NACRS) and Discharge Abstract Database (DAD) by the presence of an ICD-10 diagnostic code for TBI. The NACRS is a mandated data collection system that collects ED and ambulatory care data. Up to ten reasons for each visit to an ED in Ontario are included in the database. A reabstraction study of NACRS data compared with 7500 charts from 15 hospitals in 2004 to 2005 found good agreement in injury between NACRS and chart coding [28]. The DAD contains all acute care hospital admissions and includes demographic and clinical information on all hospital admissions and discharges, including transfers and deaths, using standard diagnosis and procedure/intervention codes. A reabstraction study of the DAD indicated good agreement for non-clinical variables, moderate to substantial agreement for the most responsible diagnosis, and good specificity of TBI codes [29, 30]. Residents of Ontario have universal access to healthcare and the province of Ontario accounts for approximately 40 % of the Canadian population with 25 % of the population were aged 19 years and under (n = 21,846,598) during the study period [31].

### Sample and case definition

Traumatic brain injury in children and youth were identified using three case definitions (please see Table 1):

**Table 1:** Case definitions

Case Definition	ICD-10 Diagnostic Codes
‘Unspecified’ Only case definition	S09.7, S09.8, S09.9
Restricted Case Definition	S01, S02.0, S02.1, S02.3, S02.7, S02.8, S02.9, S04.0, S06, S07, T01.0, T02.0, T04.0, T06.0, T90.1, T90.2, T90.4, T90.5, T90.8, T90.9
Inclusive Case Definition	S01, S02.0, S02.1, S02.3, S02.7, S02.8, S02.9, S04.0, S06, S07, S09.7, S09.8, S09.9, T01.0, T02.0, T04.0, T06.0, T90.1, T90.2, T90.4, T90.5, T90.8, T90.9

1. An inclusive case definition that included ‘unspecified injury to the head’ diagnostic codes: this was the Centers for Disease Control and Prevention (CDC) case definition for TBI related deaths. While this case definition is used to identify mortality many studies continue to utilize this case definition to identify cases in the ED and hospital (e.g.,12). This case definition includes open wound of the head (S01), fracture of the skull and facial bones (S02.0, S02.1, S02.3, S02.7, S02.8, S02.9), injury to optic nerve and pathways (S04.0), intracranial injury (S06), crushing injury of head (S07), other unspecified injuries of head (S09.7, S09.8, S09.9), open wounds involving head and neck (T01.0), fractures involving head and neck (T02.0), crushing injuries involving head and neck (T04.0), injuries of brain and cranial nerves with injuries of nerves and spinal cord at neck level (T06.0), and sequelae of injuries of head (T90.1, T90.2, T90.4, T90.5, T90.8, T90.9) [5].
2. A restricted case definition that excluded those with ‘unspecified injury to the head’ diagnostic codes. This was derived by excluding S09.7 (multiple injuries of head), S09.8 (other specified injuries of the head), and S09.9 (unspecified injury of the head) codes from the inclusive case definition.
3. The ‘unspecified injury to the head’ only case definition (thereafter referred to as the ‘unspecified’ only case definition) was those with a S09.7, S09.8, or S09.9 ICD-10 diagnostic code only.

### Variables

Demographic variables included age and sex. Children and youth aged 19 years and under were categorized into four different age groups – 0 to 4 years (infants), 5 to 9 years (children), 10 to 14 years (youth), and 15 to 19 years (older adolescents). These age categories reflect commonly used age groups in the current TBI literature for children and youth [9], including Statistics Canada [31] and World Health Organization [32].

Clinical variables included the presence of a psychiatric comorbidity, length of stay (LOS) in acute care, and special care days. LOS in acute care was defined as the number of days between admission and discharge. Special care days were defined as the cumulative number of days spent in all intensive care units.

Intention of injury and mechanism of injury variables were classified according to the CDC external cause of injury matrix [33]. Intention of injury included unintentional, suicide, assault, and undetermined/other. Mechanisms of injury were categorized into fall, motor vehicle collisions (MVC), struck by or against, and other. Other/unspecified injuries include causes such as overexertion, cut/pierce, drowning, natural/environmental, and suffocation.

Discharge disposition from acute care included death in acute care, home, home with support services, inpatient rehabilitation, complex continuing care (CCC), long term care (LTC), and transferred to another inpatient setting.

### Analyses

Episodes of care were used to determine the number and rate of healthcare utilization. Episodes of care included ED visits not admitted, ED visits with admissions, or admissions whereby a diagnostic code was not captured during an ED visit. The rationale for looking at episode of care for the number and rates analysis is because a patient may not have a TBI or ‘unspecified’ diagnosis when admitted to the ED. By linking the DAD to the NACRS via a scrambled health card number, this paper captured these patients and ensured that each episode was only captured once. This method of analysis has been shown to provide a more accurate description of the utilization of healthcare services [8]. Fiscal years 2003/04 to 2009/10 were examined for TBI episodes of care.

Patient level analysis was used to examine patient characteristics, intention and mechanisms of injury, and discharge disposition between fiscal year 2004/05 and 2009/10. This analysis captured only the first hospitalization, as a readmissions profile may differ from the initial admission. The examination of the first hospitalization for intention and mechanism of injury allowed for information on primary prevention (vs. secondary prevention with a readmissions profile). Direct age-standardized rates were generated and descriptive analyses were conducted to profile similarities and differences among hospitalized children and youth using TBI the three case definitions.

### Ethics statement

Ethics approval was obtained and received from the Toronto Rehabilitation Institute, University Health Network and administrative approval was obtained from the University of Toronto.

## Results

### TBI episodes of care

Between fiscal years 2003/04 and 2009/10, the rate of TBI episodes of care identified by the inclusive case definition (2,667.2 per 100,000) was 1.65 times higher than the rate of TBI identified by the restricted case definition (1,613.3 per 100,000). Almost 40 % of all cases identified by the inclusive case definition were ‘unspecified’ cases and, by age and sex, almost 50 % of TBI cases among youth and girls aged 10 – 19 years were coded with ‘unspecified’ (please see Table 2).

**Table 2:** Episodes of Care per 100,000 Children and Youth Aged 19 Years and Under in Ontario, Canada, 2003/04 – 2009/10

2003/04-2009/10	Overall			0-4			5-9			10-14			15-19		
	Inclusive	Restricted	Unspecified	Inclusive	Restricted	Unspecified	Inclusive	Restricted	Unspecified	Inclusive	Restricted	Unspecified	Inclusive	Restricted	Unspecified
n	582,689	352,458	230,231	222,764	134,250	88,514	130,401	88,256	42,145	100,780	53,965	46,815	128,744	75,987	52,757
rate	2,667.2	1,613.3	1,053.9	4,618.1	2,783.1	1,835.0	2,511.2	1,699.6	811.6	1,742.6	933.1	809.5	2,129.1	1,256.6	872.5
Males															
n	388,367	244,711	143,656	136,429	85,969	50,460	86,286	59,425	26,861	71,776	39,806	31,970	93,876	59,511	34,365
rate	3,472.0	2,187.7	1,284.3	5,510.2	3,472.2	2,038.0	3,249.6	2,238.0	1,011.6	2,430.5	1,347.9	1,082.6	3,027.1	1,918.9	1,108.1
Females															
n	194,322	107,747	86,575	86,335	48,281	38,054	44,115	28,831	15,284	29,004	14,159	14,845	34,868	16,476	18,392
rate	1,822.7	1,010.7	812.1	3,677.3	2,056.4	1,620.8	1,738.5	1,136.2	602.3	1,024.9	500.3	524.6	1,183.7	559.3	624.4

### Patient characteristics

The inclusive case definition identified 8,837 hospitalized children and youth while the restricted case definition identified 6,500 TBI patients between fiscal year 2004/05 and 2009/10. Among the population of TBI identified by the inclusive case definition, 34.1 % were older adolescents, 30.5 % were infants, 19.3 % were youth, and 16.1 % were children. However, among the TBI population identified using the restricted case definition, almost 40 % were older adolescents and 26.5 % were infants. Among the ‘unspecified’ only cases, 41.5 % were infants. The sex distribution of the TBI populations across different case definitions was similar (66.8 % vs. 68.0 % males) (please see Tables 3 and 4).

**Table 3:** Characteristics of children and youth by case definition, 2004/05 to 2009/10

Characteristics	Inclusive Case Definition (n = 8,837) n (Col %)	Restricted Case Definition (n = 6,500) n (Col%)	‘Unspecified’ Cases ONLY (n = 2,337) n (Col%)
Age Groups			
0-4	2,692 (30.5)	1,723 (26.5)	969 (41.5)
5-9	1,426 (16.1)	1,035 (15.9)	391 (16.7)
10-14	1,703 (19.3)	1,269 (19.5)	434 (18.6)
15-19	3,016 (34.1)	2,473 (38.0)	543 (23.2)
Sex			
Males	5,901 (66.8)	4,418 (68.0)	1,483 (63.5)
Females	2,936 (33.2)	2,082 (32.0)	854 (36.5)
Length of Stay (Days)			
1-2	4,925 (55.7)	3,218 (49.5)	1,707 (73.0)
3-5	2,252 (25.5)	1,813 (27.9)	440 (18.8)
6-11	885 (10.0)	769 (11.8)	116 (5.0)
12+	774 (8.8)	700 (10.8)	74 (3.2)
Psychiatric Comorbidity			
Yes	477 (5.4)	347 (5.3)	130 (5.6)
No	8,350 (94.6)	6,153 (94.7)	2,207 (94.4)
Special Care Days			
Yes	1,638 (18.5)	1,440 (22.2)	198 (8.5)
No	7,199 (81.5)	5,060 (77.8)	2,139 (91.5)
Intention of Injury			
Unintentional	7,955 (91.4)	5,798 (89.2)	2,157 (94.0)
Suicide	42 (0.5)	29 (0.4)	13 (0.6)
Assault	677 (7.8)	558 (8.6)	119 (5.2)
Undetermined/Other	34 (0.4)	28 (0.4)	6 (0.3)
Mechanism of Injury			
Fall	3,325 (38.2)	2,153 (33.1)	1,172 (51.1)
Motor Vehicle Collision	1,833 (21.1)	1,494 (23.0)	339 (14.8)
Struck By/Against	1,595 (18.3)	1,184 (18.2)	411 (17.9)
Other	1,955 (22.5)	1,582 (24.3)	373 (16.3)
Discharge Disposition			
Home	7,547 (85.4)	5,395 (83.0)	2,152 (92.1)
Home with Support	416 (4.7)	353 (5.4)	63 (2.7)
Rehabilitation	181 (2.1)	165 (2.5)	16 (0.7)
CCC/LTC	53 (0.6)	NR	<5
Transferred	471 (5.3)	NR	NR
Death	169 (1.9)	78 (1.2)	91 (3.9)

NR = not reportable due to small cell size

**Table 4:** Comparison of select patient characteristics by case definition, 2004/05-2009/10

Characteristics	Inclusive Case Definition (n = 8,837) n (Col %)	Restricted Case Definition (n = 6,500) n (Col%)	p-value
Demographic Characteristics			
Age 0–4 Years	2,692 (30.5)	1,723 (26.5)	<.0001
Age 5–9 Years	1,426 (16.1)	1,035 (15.9)	.7384
Age 10–14 Years	1,703 (19.3)	1,269 (19.5)	.7119
Age 15–19 Years	3,016 (34.1)	2,473 (38.0)	<.0001
Females	2,936 (33.2)	2,082 (32.0)	.1238
Clinical Characteristics			
LOS 1–2 Days	4,925 (55.7)	3,218 (49.5)	<.0001
LOS 12+ Days	774 (8.8)	700 (10.8)	<.0001
Psychiatric Comorbidity	477 (5.4)	347 (5.3)	.8877
Special Care Days	1,638 (18.5)	1,440 (22.2)	<.0001
Intention of Injury			
Unintentional	7,955 (91.4)	5,798 (89.2)	<.001
Assault	677 (7.8)	558 (8.6)	<.05
Mechanism of Injury			
Fall	3,325 (38.2)	2,153 (33.1)	<.0001
Motor Vehicle Collision	1,833 (21.1)	1,494 (23.0)	<.001
Struck By/Against	1,595 (18.3)	1,184 (18.2)	.8080
Other	1,955 (22.5)	1,582 (24.3)	<.01
Discharge Destinations			
Home	7,963 (90.1)	5,748 (88.4)	<.0001
Non-Home	705 (8.0)	674 (10.4)	<.0001
Death	169 (1.9)	78 (1.2)	<.001

The populations identified by the restricted and inclusive case definitions differed significantly with regards to LOS and special care days. Specifically, a significantly higher proportion of patients identified with the inclusive case definition stayed in acute care for less than three days (p < .0001; 55.7 % vs. 49.5 %) while a significantly higher proportion of the TBI population identified by the restricted case definition stayed in acute care for 12 days or longer (p < .0001; 8.8 % vs. 10.8 %) and spent at least one day in intensive care units (p < .0001; 18.5 % vs. 22.2 %). The inclusive and restricted case definitions did not differ significantly in the proportion of patient with psychiatric comorbidities (5.4 % vs. 5.3 %) (please see Tables 3 and 4).

### Intention and mechanism of injury

The population identified using the inclusive and restricted case definitions differed significantly in injury due to assaults (p < .05; 7.8 % vs. 8.6 %), falls (p < .0001; 38.2 % vs. 33.1 %) and MVC (p < .001; 21.1 % vs. 23.0 %). The majority of ‘unspecified’ only cases were injured unintentionally (94.0 %) with approximately half due to falls (51.1 %) (please see Tables 3 and 4).

### Discharge destinations

Among those discharged from acute care alive, a significantly higher proportion of TBI patients identified by the inclusive case definition were discharged home with or without support services (p < .0001; 90.1 % vs. 88.4 %) while a significantly higher proportion of TBI patients identified using the restricted case definition were discharged to other healthcare settings such as rehabilitation, CCC, LTC, or transferred (p < .0001; 8.0 % vs. 10.4 %). A significantly higher proportion of TBI patients identified by the inclusive case definition died in acute care (p < .001; 1.9 % vs. 1.2 %); analyses of the ‘unspecified’ cases showed that 3.9 % of patients diagnosed with ‘unspecified injury to the head’ died in acute care (please see Tables 3 and 4).

## Discussion

This is the first study, to the best of our knowledge, to compare pediatric TBI case definitions with and without ‘unspecified’ diagnostic codes using population based data in Ontario, Canada. Findings from this study revealed significant differences in the number and rate, healthcare utilization, and causes of injury when including ‘unspecified’ cases in the TBI case definition. First, up to 50 % of TBI episodes of care had an ‘unspecified’ diagnostic code, with highest rates among the infants. From a Canadian context, the ICD-10 S09.9 diagnostic code is to be assigned when “altered state of awareness, altered cognition, altered mentation, altered state of consciousness, and Glasgow Coma Scale of 3 – 12” are not documented in the record [34]. These conditions are difficult to detect in the pediatric population due to limited communication abilities and therefore, explains the high proportion of infants with TBI diagnosed with an ‘unspecified’ code. It has been suggested that the inclusion of the ‘unspecified’ diagnostic codes over time has resulted in misclassification and lower specificity of the code for TBI [18, 35]. As such, education in the importance of accurate coding in healthcare administrative data is important, particularly as 41.5 % of hospitalized infants were coded with an ‘unspecified’ code. From a prevention perspective, the inclusion of ‘unspecified’ diagnostic codes may present an opportunity to prevent mTBI. Nonetheless, the validity of these ‘unspecified’ diagnostic codes in capturing a brain injury should be explored.

Second, the inclusion of ‘unspecified’ diagnostic codes in the TBI case definition was significantly associated with shorter LOS in acute care, fewer children and youth in intensive care units, discharge home post-acute care among those discharged alive, and death in acute care. Conversely, excluding ‘unspecified’ diagnostic codes revealed a significantly higher proportion of patients staying in acute care for 12 days or longer, in intensive care units, and discharged to or transferred to other healthcare settings post-acute care, including rehabilitation, CCC, or LTC. As such, the use of a case definition for TBI that includes ‘unspecified’ diagnostic codes (i.e., the inclusive case definition) presented suggests the TBI population is not heavy users of healthcare services while excluding ‘unspecified’ diagnostic codes from the TBI case definition (i.e., the restricted case definition) suggests that children and youth with TBI require additional healthcare services post-discharge from acute care. Further, given that longer LOS is an indicator of more severe injuries, including the ‘unspecified’ cases into the TBI case definition may dilute the severity of TBI. Therefore, from the perspective of resource allocation, planning, and preparation of healthcare services for children and youth with TBI, the decision to include or exclude ‘unspecified’ cases from the TBI case definition can have significant impact on the care and services that this vulnerable population receives. Findings from this paper highlight the importance of carefully considering and identifying the goal of the research at the onset. It also provides evidence that patients diagnosed with ‘unspecified injury to the head’ diagnostic codes are likely those with less severe injury that require less healthcare services. This information should be taken into consideration when interpreting findings in this paper, as well as available data in the literature, especially when used for decision-making and resource allocation.

Finally, the intention and mechanism of injury differed significantly when including/excluding ‘unspecified’ diagnostic codes in the TBI case definition. Specifically, the TBI population identified by the restricted case definition was significantly more likely to be injured through assaults and MVC while the inclusion of ‘unspecified’ diagnostic code in the TBI case definition was significantly associated with unintentional injuries and falls. Again, from the prevention perspective, this may have significant impact in directing prevention efforts and allocating funds for specific prevention strategies. While excluding ‘unspecified’ cases will increase the specificity of the case definition for identifying TBI cases, it has been suggested that excluding these diagnostic codes may result in missed cases and underestimating the number of cases with a TBI [18]. Given the consequences of even a mTBI, the inclusion of ‘unspecified’ diagnostic codes may be beneficial, at least for the children and youth population, in preventing unintentional injuries and falls. Further, from the perspective of prevention efforts, it is preferred to overestimate rather than underestimate.

While up to 50 % of TBI episodes of care identified with the inclusive case definition in this study had an ‘unspecified injury to the head’ diagnostic code, it is acknowledged that this case definition also included diagnostic codes that may not necessarily reflect a TBI (e.g., open wound of head, facial fractures, crushing injury of the skull). Data from the first surveillance database that captured acquired brain injuries across the continuum of healthcare in Ontario, Canada, provided information on the frequency of some commonly used diagnostic codes to define TBI [36]. Identified through stakeholder consultation, the following codes were examined – “fracture of the skull (S02.0, S02.1, S02.7, S02.89, S02.9), intracranial injury excluding those with skull fracture (S06, S09.7, S09.8, S09.9), late effects of injuries, poisonings, toxic effects & other external causes (T90.2, T90.5, T90.8, T90.9, T96, T97, T98.2), and certain traumatic complications and unspecified injuries (S09.0, S09.1, S09.2)” [36]. This report showed that 79 % of the records identified with these codes were diagnosed with “intracranial injury excluding those with skull fracture” while 16 % were diagnosed with “fracture of the skull”. Approximately 5 % were diagnosed with the remaining two categories, suggesting that these diagnostic codes do not add many cases to the case definition [36]. A recent systematic review on the range of ICD-10 codes used to define pediatric TBI corroborates with findings from the scientific report described [9]. Specifically, this review found that the majority of articles that met inclusion criteria included diagnostic codes related to facial fractures and intracranial injury, as well as S01 (open wound to the head), S04.0 (injury to optic nerve and pathways), and S07 (crushing injury of the head). There is currently evidence in the literature that supports the inclusion of diagnostic codes related to facial fractures, injury to optic nerve and pathways, and crushing injury of the head in the case definition for TBI. For example, a TBI among individuals with facial fractures is very common, including an increased risk for fractures of the orbital floor (S02.3), malar maxillary bones (S02.4), and the mandible (S02.6) [37–42]. There is also evidence in the literature that supports a relationship between optic nerve injury and a brain injury [43–45]. Similarly, even though crushing injuries to the skull may result in less severe neurological damage, brain injury can still occur if the forces applied are greater than the tolerance of the cranium [46–48]. As such, in the absence of validation studies on these codes, the literature suggests that the diagnostic codes related to facial fractures, injury to optic nerve and pathways, and crushing injury to the head, which are included even in the restricted case definition for this paper, are likely capturing a TBI.

However, what is unclear is the inclusion of open wound of the head (S01) codes. In the Canadian context, “open wounds include animal bite, cuts, lacerations, avulsion of skull, and subcutaneous tissue and puncture wounds with or without penetrating foreign body. They do not include traumatic amputation or avulsions that involve deeper tissue” [34]. It is unclear, from this definition, whether these codes would capture a TBI. However, when applying the definition of TBI from the CARF [1], it suggests that open wounds should be included, as a TBI includes “open head injuries” that include “other penetrating injuries”. Additional research is required to determine the circumstances in which these codes are used in the clinical setting and the extent to which the inclusion diagnostic codes S01 affect the numbers and outcomes of the TBI population, especially as there is currently no data demonstrating the frequency in which these codes are used.

Limitations associated with the use of healthcare administrative data must be recognized, including coding issues and missed cases, particularly for this paper, as it relies on the coding of the diagnostic codes included for this study. While Juurlink and colleagues assessed found good agreement for S06, intracranial injury, diagnostic codes [30], there are currently no validation studies on the other diagnostic codes used to define TBI. Importantly, it is unclear the extent to which the ‘unspecified injury to the head’ diagnostic codes were true or false positives and thus, the rate of TBI episodes of care presented in this paper may not reflect an accurate rate. However, the goal of presenting information on TBI episodes of care in this paper was to illustrate the extent to which ‘unspecified injury to the head’ diagnostic codes affect these numbers. As such, information from this study can facilitate discussion with evidence that can be used to assist efforts to reach the most appropriate and accurate case definition for TBI. Further, given the nature of the data, this study only included individuals who sought medical attention. It is acknowledged that many milder TBI cases do not seek medical treatment but can still experience on-going problems [19]. This study attempted to capture more milder TBI that seek medical treatment by identifying cases that present in the emergency department by using episodes of care to assess the burden of healthcare services. Current data that use only hospitalization data are likely to capture more moderate to severe injuries. However, milder TBI are likely to present in the emergency department and this diagnosis may not be made in the acute care setting. As such, linking the DAD to the NACRS ensured that cases that are not coded as a TBI in the DAD were captured in the NACRS and that double counting did not occur. Finally, information on discharge destinations from acute care, which were used to assess the healthcare needs of this population, was based on coded information in the databases rather than actual linkage of cases across the continuum of care. Additional variables of interest that may provide more information on the outcome of this population are not well coded or are unavailable (e.g., functional status, detailed severity of injury information).

Nevertheless, this is the first population-based study that compared children and youth with TBI with and without ‘unspecified’ diagnostic codes to elucidate how these ‘unspecified’ codes affect the number and profile of the TBI population. The province of Ontario in Canada has publicly funded healthcare. Therefore, this study captured every single resident of Ontario that met the inclusion criteria of this paper between fiscal years 2003/04 and 2009/10 in any emergency department or the acute care setting in Ontario. Efforts to identify the optimal case definition for TBI in children and youth are encouraged to explore the sequelae of injury code, as it has been suggested that the inclusion of sequelae codes allows for the capturing of patients that were missed in their first admission [49]. As such, separate analyses on this population can advise future work on reaching consensus on the case definition for this population. Even a mTBI can have long lasting consequences [19] and thus, capturing children and youth suffering from late effects of injuries may prevent re-injury and assist in assessing the burden of TBI on the healthcare system. Further, many studies have identified retinal hemorrhage as a predictor of inflicted TBI in infants [50], including shaken baby syndrome [51, 52] and abusive head trauma [45, 53]. It has been stated that retinal hemorrhage is present in 50 % to 100 % of cases, often clinches the diagnosis of shaken baby syndrome, and that retinal hemorrhage can rarely occur without intracranial hemorrhage or cerebral edema [54–56]. As such, the inclusion of retinal hemorrhage ICD-10 codes H35.6 should be explored in identifying infants and children with TBI.

## Conclusion

This paper serves to provide the foundation for future work on reaching an appropriate and accurate case definition to define TBI in children and youth that can be used worldwide. It provided evidence that the number and rate, healthcare use, and intention and mechanism of injury differed significantly when including/excluding ‘unspecified’ diagnostic codes in the TBI case definition for children and youth. The evidence and suggestions for ICD-10 codes presented in this study can improve and encourage the use of these codes in a more standard way internationally, which can assist in informing the needs of children and youth with TBI and efforts to improve the quality of life of this population through adequate healthcare services and support post-hospitalization.

## Abbreviations

CCC: Complex continuing care
CDC: Centers for disease control and prevention
CIHI: Canadian institute for health information
DAD: Discharge abstract database
ED: Emergency department
ICD: International classification of diseases
ICD-9: International classification of diseases version 9
ICD-10: International classification of diseases version 10
LOS: Length of stay
LTC: Long-term care
mTBI: Mild traumatic brain injury
MVC: Motor vehicle collision
NACRS: National ambulatory care reporting system
TBI: Traumatic brain injury
